# Rapid Evolution of Parasite Resistance in a Warmer Environment: Insights from a Large Scale Field Experiment

**DOI:** 10.1371/journal.pone.0128860

**Published:** 2015-06-02

**Authors:** Fernando Mateos-Gonzalez, L. Fredrik Sundström, Marian Schmid, Mats Björklund

**Affiliations:** 1 Department of Animal Ecology, Evolutionary Biology Centre, Uppsala University, Uppsala, Sweden; 2 Department of Fish Ecology, Limnological Institute, Universität Konstanz, Konstanz, Germany; Institut Pasteur, FRANCE

## Abstract

Global climate change is expected to have major effects on host-parasite dynamics, with potentially enormous consequences for entire ecosystems. To develop an accurate prognostic framework, theoretical models must be supported by empirical research. We investigated potential changes in host-parasite dynamics between a fish parasite, the eyefluke *Diplostomum baeri*, and an intermediate host, the European perch *Perca fluviatilis*, in a large-scale semi-enclosed area in the Baltic Sea, the Biotest Lake, which since 1980 receives heated water from a nuclear power plant. Two sample screenings, in two consecutive years, showed that fish from the warmer Biotest Lake were now less parasitized than fish from the Baltic Sea. These results are contrasting previous screenings performed six years after the temperature change, which showed the inverse situation. An experimental infection, by which perch from both populations were exposed to *D*. *baeri* from the Baltic Sea, revealed that perch from the Baltic Sea were successfully infected, while Biotest fish were not. These findings suggest that the elevated temperature may have resulted, among other outcomes, in an extremely rapid evolutionary change through which fish from the experimental Biotest Lake have gained resistance to the parasite. Our results confirm the need to account for both rapid evolutionary adaptation and biotic interactions in predictive models, and highlight the importance of empirical research in order to validate future projections.

## Introduction

Global climate change is likely to become one of the biggest challenges we will face over the next century and, as such, a tremendous effort is being applied to advance the emerging field of climate change ecology [1]. However, the complexity of the problem still exceeds the capacity of the current models to predict future impacts. To overcome this limitation, models require validation by comparing their projections to actual observations and to results from empirical research [1]

One of the major concerns derived from climate warming is the potential impact on host-parasite dynamics, which could lead to an increase in infectious diseases, affecting whole ecosystems [2–5]. The evolution of host-parasite population dynamics is difficult to predict even under relatively stable conditions, given the complexity of the interactions. Changing conditions can add further layers of uncertainty, as host-parasite interactions are highly sensitive to environmental changes [3,6]. This is especially relevant in aquatic ecosystems, where changes in temperature, increased pollution associated with flooding, and carbon dioxide acidification are expected to result in faster and more acute alterations than in other environments [7].

In this study, we aim to gain empirical insights in host-parasite population dynamics in a changing environment by taking advantage of a large scale, long-term “natural” experiment.

The Biotest Lake outside the Forsmark nuclear power plant (Forsmark, Sweden) is a part of the Baltic Sea artificially that was artificially enclosed in 1977. As a result, fish inside the lake became isolated from the surrounding population. Warmed water from the cooling system of the power plant has been continuously discharged into the Biotest Lake since 1980, resulting in a year-round increased water temperature, 6–10°C higher than the surrounding Baltic Sea [8]. The influx from the power plant also prevents any ice coverage in the Biotest Lake, which would otherwise be present during 3–5 months in the winter. The gratings that prevented fish migration between the enclosure and the surrounding sea were removed in 2004, once again enabling gene flow between fish sub-populations [9]. However, the flow out from the Biotest Lake still provides an almost constant barrier against fish migration into the enclosure.

The European perch, *Perca fluviatilis* Linnaeus 1758, is an abundant fish species both inside and outside the Biotest Lake, and a common intermediate host for the trematode parasite *Diplostomum baeri* Dubois 1937. The prevalence and infection intensity of this parasite in perch was studied in the early years after the population split [8], and was found to be significantly higher in the warmer isolated area than in the surrounding water. Now, three decades later, there are indications that the host-parasite dynamics have changed again. First, the population of the mollusc *Radix balthica*, an intermediate host for the parasite *D*. *baeri*, is almost extinct in the Biotest Lake and has been replaced with the exotic snail *Potamopyrgus antipodarum* [10]. This exotic snail is not yet a known host for *D*. *baeri*, and thus the numbers of intermediate hosts that could be maintaining the parasite inside the Biotest Lake must be significantly lower. At the same time, since the construction of the enclosure, there has been strong selection on MHC in the perch inside the Biotest Lake, both in terms of number of loci and allelic richness [11], which could be an indication of a change in parasite resistance [12,13].This situation provides an excellent opportunity to study the effect of a drastically changed environmental factor, water temperature, on the evolution of host-parasite interactions, in a single population recently split into two.

Specifically, our aims were to examine if the altered conditions have produced a change in prevalence and/or intensity of infection, and if these potential variations in infection have led to (or might have been caused by) a difference in parasite resistance. We investigated these potential changes in host-parasite dynamics by 1) determining prevalence and intensity of infection in the perch population, both from the warmer Biotest Lake and from the surrounding Baltic Sea natural population, in two consecutive years (2013–2014), and 2) performing an artificial infection experiment, by which perch from both the Biotest Lake and the Baltic Sea were exposed to *D*. *baeri* collected from the Baltic Sea. Based on the data on snail abundance, we predicted that the prevalence and infection intensity would be lower in the Biotest Lake compared to the Baltic Sea population. Under this condition, we could expect the current population of Biotest perch to be potentially naïve to the parasite, and we predicted that, in that case, an experimental infection should produce a higher infection intensity and prevalence in Biotest fish than in the Baltic population.

## Materials and Methods

The Biotest Lake is a semi-enclosed lake in the Baltic Sea adjacent to the nuclear power plant at Forsmark (east coast of Sweden, 60°25’N, 18°10’ E) and covers an area of 0.9 km^2^ with a mean depth of 2.5m. Heated water from the power plant has been continuously discharged into the Biotest Lake since 1980, which has resulted in a year-round temperature in the lake about 6–10°C warmer than the surrounding Baltic Sea [8]. Water is brackish both in and outside the lake (salinity around 1‰). Apart from temperature, physical conditions between the Biotest Lake and the surrounding Baltic do not differ greatly. The outflow from the lake was screened by a 15 mm mesh size grid during 1981–2004, most likely resulting in isolation of the Biotest fish populations from the Baltic Sea population for 23 years [8,10]. In 2004, the grid was removed making it possible for larger fish to move between the lake and the surrounding Baltic Sea.

*D*. *baeri* is a trematode whose life cycle requires three different hosts; a mollusc, a fish, and a final avian host. The larval form, *cercariae*, originates through asexual reproduction from an infected snail. When the free swimming *cercariae* find a fish host, they penetrate through the skin and gills, then move to the eye and form *metacercariae* in the vitreous body. Piscivorous birds close the cycle by consuming the infected fish. The parasites mature and reproduce sexually in the intestines of the bird. Eggs are then released with the bird’s excrements. These eggs hatch as *miracidia* and in turn infect snails [14]

The European perch *Perca fluviatilis* is susceptible to infection from *D*. *baeri*. The level of infection of *D*. *baeri* was studied in the perch during 1986 and 1987[8]. In spring, the prevalence of the parasite was 100% in the Biotest Lake, and 93% in the outside Baltic Sea population, reaching 100% in both locations during summer. During this period, the intensity of infection was significantly higher in the Biotest population than in the Baltic Sea population [8]

In September 2013, we captured 135 young-of-the-year (YOY) perch by hand trawl in four different locations, two inside the Biotest Lake and two in the Baltic Sea. Sixty-five of these fish (26 individuals from the Baltic Sea and 39 individuals from the Biotest Lake) were used as a control group for the infection experiment (see below), and allowed us to estimate the natural infection intensity and prevalence both inside the Biotest Lake and in the surrounding Baltic Sea. These fish covered a size range of 47–89 mm for Baltic perch and 48–93 mm for Biotest perch.

A second sampling of 60 perch from the Baltic Sea and 60 perch from the Biotest Lake was performed in July and August 2014, in order to increase the sampling locations, and to investigate potential age effects on the infection. To do so, we sampled three more locations inside the Biotest and three in the surrounding Baltic Sea. In this second screening, we employed gill nets to be able to capture both adult and juvenile individuals. These fish covered a size range of 149–296 mm for Baltic perch and 147–309 mm for Biotest perch.

The infection experiment was carried out on the 135 YOY perch captured in September 2013. The 65 fish screened for natural infection intensity and prevalence were used as control, while the remaining 34 Biotest and 36 Baltic YOY perch were exposed to *cercariae* of *D*.*baeri*. More than 100 snails, including the species *Radix balthica* (the main potential host of *D*. *baeri* in the Forsmark region), were collected from multiple locations inside the Biotest Lake and surrounding Baltic Sea. Snails were kept under light in shallow water overnight and water was sampled in the morning and examined for *D*. *baeri cercariae*. No *cercariae* were found in the water of Biotest snails. Large numbers of *cercariae*, later identified as *D*. *baeri* by means of barcoding [15], were found in the water of snails collected in the Baltic Sea. Hence, perch were infected only with *cercariae* from the Baltic Sea and not the Biotest Lake.

The experimental infection was performed by exposing five perch at a time held in 1.5 l tanks containing ~4500 *cercariae*. The parasites were added to each experimental tank from a common recipient that contained cercariae pooled from all the snails collected in the Baltic area. By mixing cercariae originating from different snails, we aimed to expose fish to different genotypes of the parasite, to minimize differences in infection success due to differences in genotypic diversity [16]. Each group of five perch was exposed to a new sample of cercariae originating from the same common recipient. To minimize stress, exposure was limited to 10 min before fish were transferred to parasite free water. Control fish were held for the same time in parasite free tanks. All fish were then transferred to four 45 l containers held in a 1200 l tank with flow-through water, each group kept separate. To allow parasites to move from the gills and skin to the eyes, all fish were kept for 7 days until dissected.

Infection intensity and prevalence were assessed by dissecting the eyes. First fish were euthanized by an extended exposure to MS222, followed by decapitation and extraction of the intact eyes. Each eye was carefully pulled apart and the number of *metacercariae* was counted under a stereoscope. Dissections were performed by two people, consistently double-checking parasite counts throughout the observations.

Data could not be transformed to fulfill the requirements of a GLM or ANOVA (normality of error, homogeneity of variance and linearity), so the response to parasite exposure was tested with a Mann-Whitney U-test comparing infected fish with control fish, separate for Biotest and Baltic fish. The length of Biotest control fish (68 mm ± 7.8 SD) did not differ from infected Biotest fish (71 mm ± 11.2 SD; p = 0.45) and neither did Baltic control fish (65 mm ± 9.4 SD) from Baltic infected fish (68mm ± 9.3 SD; p = 0.14). To control for potential false positives or negatives during infection estimation [17], true prevalence was calculated from apparent prevalence using the function *truePrev*, in the *prevalence* package [18], in the statistical program R, version 3.0.3 [19]. This function allows the calculation of a Bayesian analysis that takes into account a flexible sensitivity (SE, the probability that a truly infected individual will test positive) and a flexible specificity (SP, or the probability that a truly non-infected individual will test negative). SE was coded to vary from 75% to 90%, whereas SP was allowed to vary from 90% to 100%. We used a more stringent criteria for SP since finding at least one parasite means that the fish was truly infected, while failing to do so does not eliminate the possibility of an infection.

Samples of *metacercariae* collected from the perch eyes were preserved in ethanol and the species were identified by genetic analysis using the barcode primers for COI and the PCR-protocol developed by Moszczynska *et al*. [15] and Behrmann-Godel [20].

All analyses were implemented in R, version 3.0.3 [19]The experimental procedures described in this study were reviewed and approved by the regional ethical committee (permit Uppsala djurförsöksetiska nämnd C104/12), and the Institute of Coastal Fisheries issued the permits to work in the described study sites.

## Results

**Fig 1:**
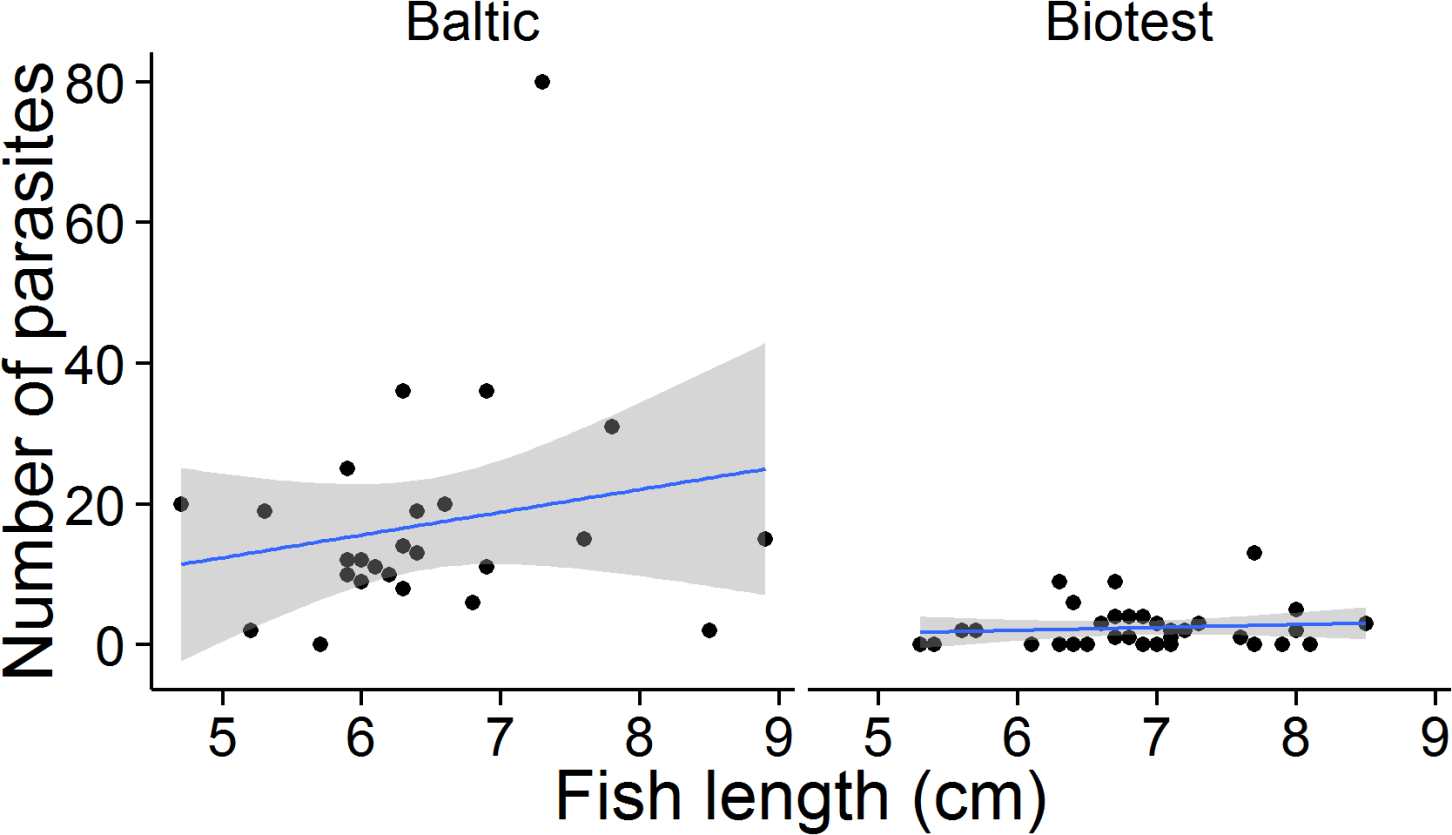
Infection intensity in 2013 of the parasite *D*. *baeri* in juvenile (YOY) perch from the Baltic Sea and the heated Biotest Lake. The line indicates best-fit and the gray area represents the 95% confident interval.

In 2013, the *true* prevalence of *D*. *baeri* in YOY fish was 77% (95% credible interval 56–97%) in the Biotest control fish, and 94% (95% credible interval 82–100%) in the Baltic control fish. There were no significant correlations between fish length and number of parasites in either of the groups (p > 0.1 in all cases), thus length was not corrected for. The natural infection intensity differed significantly between the populations (χ^2^ = 31.73, df = 2, P << 0.001, Fig 1). The intensity of infection in Baltic fish was on average 7.2 times higher than in the corresponding Biotest fish (Baltic mean = 17.2, Baltic median = 12.5, SD = 15.9, range 1–80, Biotest mean = 2.4, Biotest median = 7.5, SD = 2.9, range 1–13; Z = 5.67, P < 0.001, Mann-Whitney U-test).

**Fig 2:**
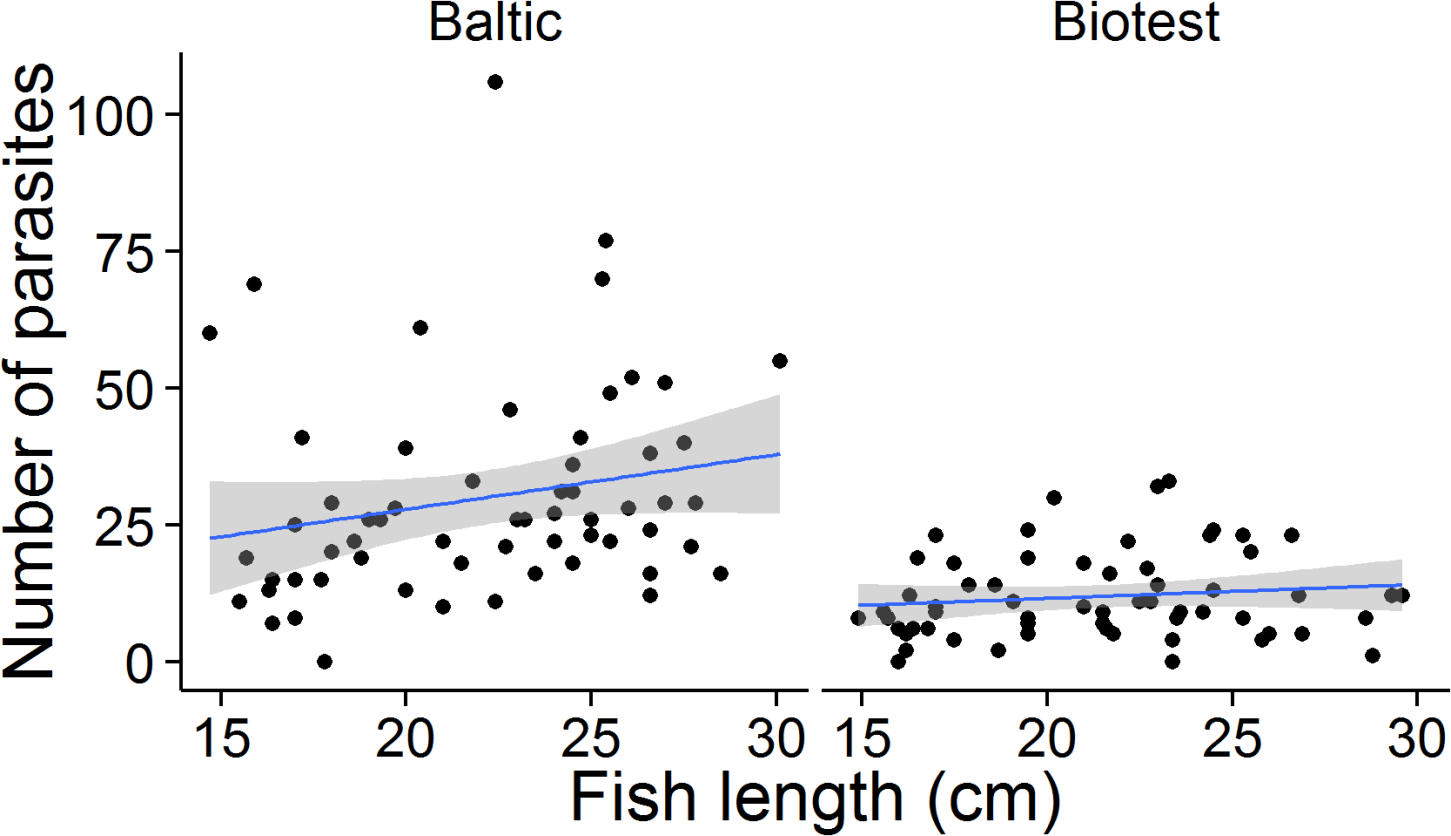
Infection intensity in 2014 of the parasite *D*. *baeri* in pre-adult and adult perch from the Baltic Sea and the heated Biotest Lake. The line indicates best-fit and the gray area represents the 95% confident interval.

In 2014, when fish from different age groups were screened, the distribution of infection intensity still differed significantly between the Biotest and Baltic fish (χ^2^ = 31.76, df = 2, p< 0.001, Fig 2). There were no differences in infection intensity between sampling locations within the Baltic (χ ^2^ = 1.17, df = 2, p = 0.558) or within the Biotest (χ ^2^ = 2.19, df = 2, p = 0.334). The *true* prevalence of *D*. *baeri* was 96% (95% credible interval 90–100%) in the Biotest fish, and 98% in the Baltic fish (95% credible interval 95–100%).

Baltic fish seem to acquire slightly more parasites as they age (Spearman rank correlation between length and number of parasites: n = 60, r = 0.32, p = 0.01), but this was not the case in the Biotest Lake (Spearman rank correlation: n = 60, r = 0.15, p = 0.25), (Fig 2).

**Fig 3:**
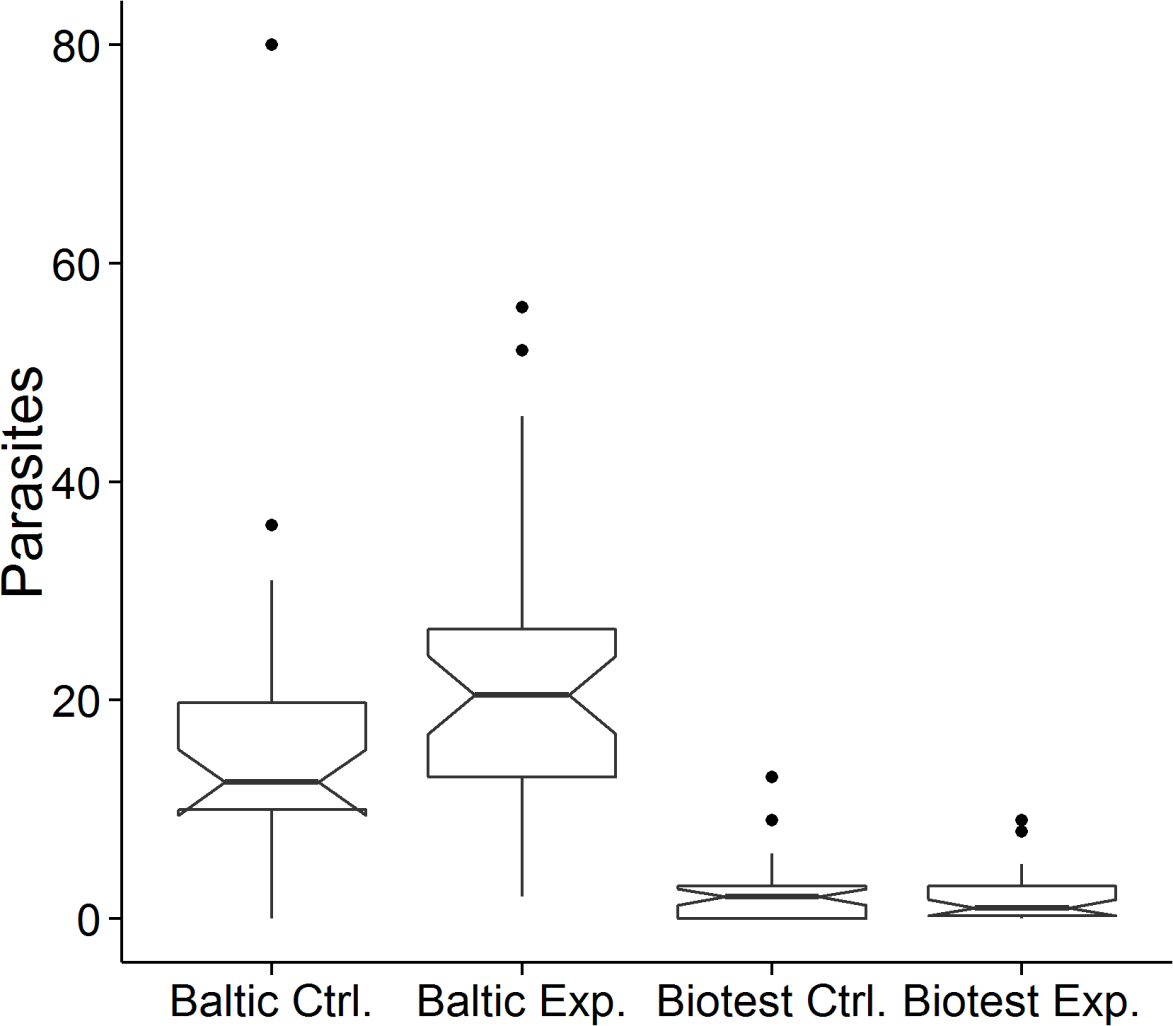
Between-groups comparison of number of parasites in control (C) and experimental (E) groups in Baltic and Biotest perch. The notched boxplot indicates the median, the 2nd and 3rd quartiles, and the range. If two boxes' notches do not overlap there is ‘strong evidence’ (95% confidence) that their medians differ [30].

The experimental exposure to parasites did not have an effect in fish from the Biotest Lake (*Z* = 0.27, *p* = 0.78), but it did in fish from the Baltic Sea, resulting in a significantly higher intensity of infection than that of the control Baltic group (*Z* = -2.37, *p* = 0.018; Fig 3). On average, the experimental infection increased the intensity of infection in the Baltic Sea fish with almost 40% (Fig 3).

## Discussion

**Fig 4:**
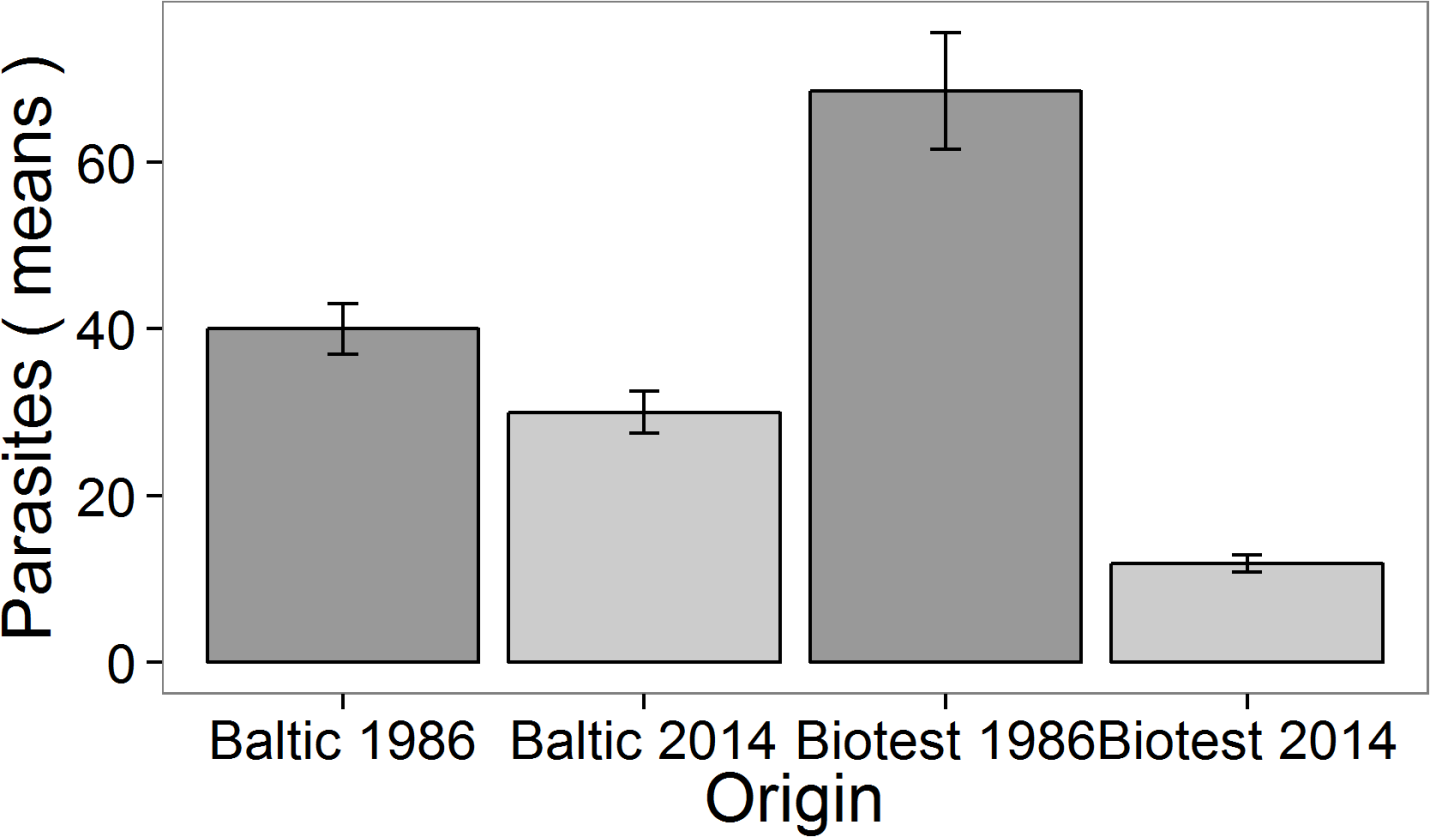
Intensity of infection in Biotest and Baltic fish in July-August 1986 [8], and July-August 2014.

Our results show that fish from the warmed Biotest Lake have a much lower intensity of *D*.*baeri* infection than fish from the surrounding natural Baltic Sea, for at least two consecutive years (2013–2014). This situation poses as a dramatic contrast to a previous studied period, that of years 1986 and 1987. Back then, less than 10 years after the enclosure was built and the water started to get warmer, Biotest fish already showed intensity levels of parasite infection that were almost twice that of Baltic fish [8], which is an expected outcome under warmer conditions [4,21]. By 2013, however, the intensity of infection in Biotest fish has been reduced by almost 80%, whereas the control Baltic population has shown only a slight decrease (Fig 4). Contrary to our predictions, Biotest fish did not suffer an increase in infection intensity after the experimental exposure to parasites, whereas Baltic fish showed significantly higher numbers of *metacercariae* than their control counterparts.

The current difference in natural infection intensity between both locations could have been caused by several reasons. Given that Biotest perch mature at an earlier age than Baltic perch [22] probably due to the difference in temperature, the higher intensity of infection observed in the Baltic fish could be explained by a potential delayed maturation of the Baltic fish immune system. However, the fact that the infection differences between Baltic and Biotest fish are maintained throughout older age groups deems this explanation unlikely.

A more plausible reason is that the recent decline in the intermediate host population, the snail *R*. *balthica* might have been hampering the parasite reproduction by disrupting its cycle, hence reducing the numbers of the parasite in the Biotest Lake. If that were the case, we could expect a lower selection pressure from the parasite on perch in the Biotest Lake. Even though, to our knowledge, the specific pathological effects of *D*. *baeri* have not been described, trematode *cercariae* are known to produce harmful and even lethal effects during migration through their fish hosts, including haemorrhage, muscle necrosis, or congested blood vessels [23] and references therein). Given the cost of maintaining an immune response, natural selection should rapidly select for individuals that reallocate resources to other uses when an immune response is less, or no longer needed [24]). According to this logic, if parasites are disappearing from the Biotest Lake, we should expect Biotest perch to suffer an increase in infection after an experimental exposure to the parasite. Furthermore, it is known that a first contact with the parasite can improve the resistance against subsequent infections [25,26]. Given that Biotest YOY control fish showed a lower prevalence of infection than their Baltic counterparts, we would have expected the more naive Biotest fish to be affected more intensely by the experimental treatment.

Our experimental infection, however, showed that Biotest fish, living in an environment seemingly free (or at least with a much lower presence) of the parasite, did not suffer an effect from the infection treatment. Baltic fish, on the other hand, did suffer a significant infection intensity increase after the infection experiment.

Recent work has shown that, under certain conditions, the experimental elimination of parasites could lead to the evolution of enhanced parasitic resistance in hosts [27]. The authors suggested that in cases where other selection pressures varied at the same time, inducing a slower life history (e.g. reduced predation pressures), an increased resistance to the parasite could emerge through pleiotropic or functional associations.

Another potential explanation to our results could be that, during the first years of warm water discharge into the lake, the higher number of parasites in the Biotest fish (which presumably increased due to the higher temperature in the Biotest Lake [4,21] resulted in a strong selection on Biotest fish to develop immunity to the parasite. This outcome is strongly supported by the data on changes in MHC in the same population [11]. At the same time, whereas the perch in the Biotest were genetically isolated from the Baltic perch population, parasites from the Baltic Sea could possibly still enter the Biotest Lake from bird transportation. Thus, Biotest perch may initially have experienced strong selection by repeated infections by the same parasite genotype, while the parasite could not co-evolve with the Biotest fish because it was also interacting with the Baltic Sea perch across generations. The snail *R*. *balthica*, now mostly displaced by the exotic *P*. *antipodarum*, could have also suffered from the initially large numbers of the parasites. Intense episodes of *cercariae* output are known to be lethal to second intermediate hosts, potentially leading to mass mortality and local extinction [28]. The rapid decline of the snail could have greatly contributed to the eventual escape of the Biotest perch from the host-parasite race.

In conclusion, our results describe how an increased temperature has potentially aided a dramatic change in host-parasite dynamics in about three decades, due to ecological mismatches between several interacting species. In particular, the fast change in parasite resistance observed in the Biotest perch adds to the uncommon evidence of parasite-mediated selection in natural populations [29]. These results have direct implications for consequences of global climate change, as they show that fast environmental changes can lead to equally rapid evolutionary responses. Finally, as advocated by recent theoretical approaches [5], our results confirm the need to account for rapid evolutionary adaptation and biotic interactions in predictive models, and highlight the importance of empirical research in order to validate future projections.

## Supporting Information

S1 Dataset (XLSX)
